# *N*-Cyclo­hexyl-*N*-propyl­benzene­sulfonamide

**DOI:** 10.1107/S1600536809046650

**Published:** 2009-11-21

**Authors:** Zeeshan Haider, Islam Ullah Khan, Muhammad Zia-ur-Rehman, Muhammad Nadeem Arshad

**Affiliations:** a Department of Chemistry, Government College University, Lahore 54000, Pakistan; b Applied Chemistry Research Centre, PCSIR Laboratories Complex, Ferozpure Road, Lahore 54600, Pakistan

## Abstract

The title compound, C_15_H_23_NO_2_S, synthesized by *N*-methyl­ation of cyclo­hexyl­amine sulfonamide with propyl iodide, is of inter­est as a precursor to biologically active sulfur-containing heterocyclic compounds. The cyclo­hexyl ring exists in the chair form and the dihedral angle between the ring plane of the benzene ring and that of the cyclo­hexyl ring is 50.13 (9)°.

## Related literature

For the synthesis of related mol­ecules, see: Kayser *et al.* (2004 ▶); Zia-ur-Rehman *et al.* (2006 ▶, 2009 ▶). For the biological activity of sulfonamides, see: La Roche & Co (1967 ▶); Rough *et al.* (1998 ▶); Gennarti *et al.* (1994 ▶). For related structures, see: Arshad *et al.* (2008 ▶); Khan *et al.* (2009 ▶); Gowda *et al.* (2007*a* ▶,*b* ▶,*c* ▶). For bond-length data, see: Allen *et al.* (1987 ▶).
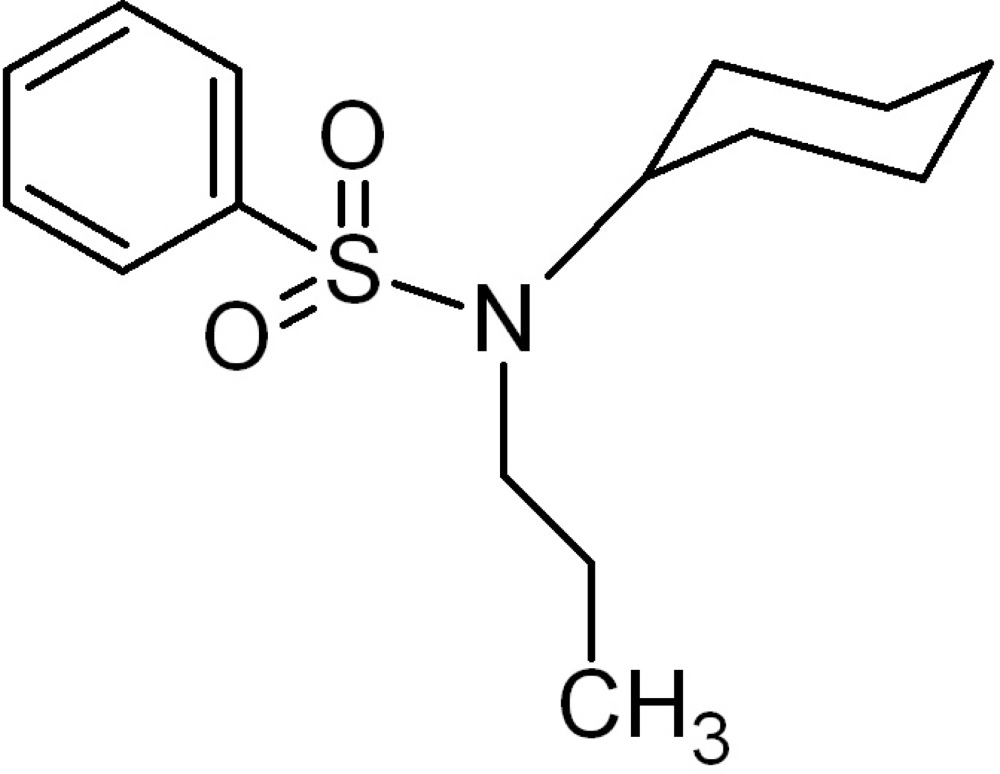


## Experimental

### 

#### Crystal data

C_15_H_23_NO_2_S
*M* *_r_* = 281.40
Monoclinic, 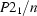

*a* = 8.5532 (3) Å
*b* = 11.6877 (4) Å
*c* = 15.4410 (5) Å
β = 90.649 (2)°
*V* = 1543.50 (9) Å^3^
*Z* = 4
Mo *K*α radiation
μ = 0.21 mm^−1^
*T* = 296 K
0.42 × 0.31 × 0.25 mm

#### Data collection

Bruker APEXII CCD area-detector diffractometer
Absorption correction: multi-scan (*SADABS*; Bruker, 2007 ▶) *T*_min_ = 0.918, *T*_max_ = 0.950
17345 measured reflections
3839 independent reflections
2475 reflections with *I* > 2σ(*I*)
*R*_int_ = 0.041

#### Refinement

*R*[*F* ^2^ > 2σ(*F* ^2^)] = 0.050
*wR*(*F* ^2^) = 0.141
*S* = 1.03
3839 reflections
173 parameters
H-atom parameters constrained
Δρ_max_ = 0.29 e Å^−3^
Δρ_min_ = −0.21 e Å^−3^

### 

Data collection: *APEX2* (Bruker, 2007 ▶); cell refinement: *SAINT* (Bruker, 2007 ▶); data reduction: *SAINT*; program(s) used to solve structure: *SHELXS97* (Sheldrick, 2008 ▶); program(s) used to refine structure: *SHELXL97* (Sheldrick, 2008 ▶); molecular graphics: *PLATON* (Spek, 2009 ▶) and *Mercury* (Macrae *et al.*, 2006 ▶); software used to prepare material for publication: *WinGX* (Farrugia, 1999 ▶) and *PLATON*.

## Supplementary Material

Crystal structure: contains datablocks I, global. DOI: 10.1107/S1600536809046650/hg2581sup1.cif

Structure factors: contains datablocks I. DOI: 10.1107/S1600536809046650/hg2581Isup2.hkl

Additional supplementary materials:  crystallographic information; 3D view; checkCIF report

## Figures and Tables

**Fig. 1.**
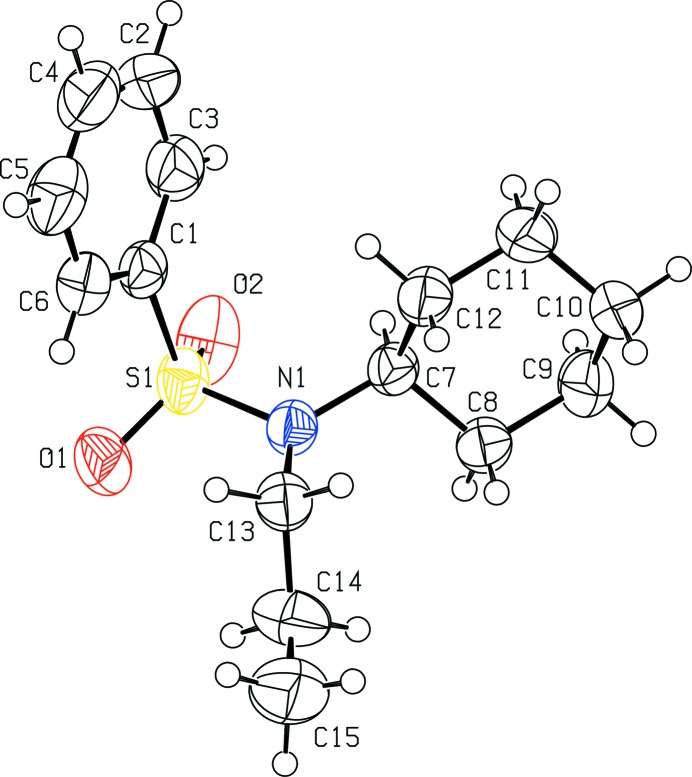
The molecular structure of (I), with displacement ellipsoids at the 50% probability level.

## supplementary crystallographic information

### Comment

Sulfonamides are well known for their enormous potential as biologically active molecules (Rough *et al.*, 1998). They are being used as anti-microbial (Kayser *et al.*, 2004), anti-convulsant (Arshad *et al.*, 2008), anti-cancer (La Roche & Co, 1967) agents and for the treatment of inflammatory rheumatic and non-rheumatic processes including onsets and traumatologic lesions (Gennarti *et al.*, 1994). In the present paper, the structure of *N*-cyclohexyl-*N*-propyl benzene sulfonamide has been determined as part of a research program involving the synthesis and biological evaluation of sulfur containing heterocyclic compounds (Zia-ur-Rehman *et al.*, 2006, 2009; Khan *et al.*, 2009). In the molecule of (I) (Scheme 1; Fig. 1), bond lengths and bond angles are almost similar to those in the related molecules (Gowda *et al.*, 2007*a*,*b*,*c*) and are within normal ranges (Allen *et al.*, 1987). The benzene ring is essentially planar while cyclohexane ring is in chair form. No significant hydrogen bond interactions are observed in the title molecule. The dihedral angle between the phenyl and cyclohexane rings is 50.13 (9)%.

### Experimental

A mixture of *N*-cyclohexylbenzene sulfonamide (1 g, 0.43 mmol), sodium hydride (0.21 g; 0.88 mmoles) and *N*, *N*-dimethylformamide (10.0 ml) was stirred at room temperature for half an hour followed by addition of propyl iodide (0.146 g; 0.86 mmoles). Stirring was continued further for a period of three hours and the contents were poured over crushed ice. Precipitated product was isolated, washed and crystallized from methanol.

### Refinement

All hydrogen atoms were refined geometrically and treated as riding on their parent atoms. The following distances were used: Aromatic C–H=0.93Å, methine C–H=0.98Å, methylene C–H=0.97Å and methyl C–H=0.96Å U(H) was set to 1.2Ueq of the parent atoms or 1.5Ueq for methyl group.

### Crystal data

**Table:**

C_15_H_23_NO_2_S	*F*(000) = 608
*M**_r_* = 281.40	*D*_x_ = 1.211 Mg m^−^^3^
Monoclinic, *P*2_1_/*n*	Mo *K*α radiation, λ = 0.71073 Å
Hall symbol: -P 2yn	Cell parameters from 4904 reflections
*a* = 8.5532 (3) Å	θ = 2.2–24.1°
*b* = 11.6877 (4) Å	µ = 0.21 mm^−^^1^
*c* = 15.4410 (5) Å	*T* = 296 K
β = 90.649 (2)°	Needles, colourless
*V* = 1543.50 (9) Å^3^	0.42 × 0.31 × 0.25 mm
*Z* = 4	

### Data collection

**Table:**

Bruker APEXII CCD area-detector diffractometer	3839 independent reflections
Radiation source: fine-focus sealed tube	2475 reflections with *I* > 2σ(*I*)
graphite	*R*_int_ = 0.041
φ and ω scans	θ_max_ = 28.3°, θ_min_ = 2.2°
Absorption correction: multi-scan (*SADABS*; Bruker, 2007)	*h* = −11→11
*T*_min_ = 0.918, *T*_max_ = 0.950	*k* = −15→15
17345 measured reflections	*l* = −20→18

### Refinement

**Table:**

Refinement on *F*^2^	Primary atom site location: structure-invariant direct methods
Least-squares matrix: full	Secondary atom site location: difference Fourier map
*R*[*F*^2^ > 2σ(*F*^2^)] = 0.050	Hydrogen site location: inferred from neighbouring sites
*wR*(*F*^2^) = 0.141	H-atom parameters constrained
*S* = 1.03	*w* = 1/[σ^2^(*F*_o_^2^) + (0.0648*P*)^2^ + 0.2752*P*] where *P* = (*F*_o_^2^ + 2*F*_c_^2^)/3
3839 reflections	(Δ/σ)_max_ = 0.001
173 parameters	Δρ_max_ = 0.29 e Å^−^^3^
0 restraints	Δρ_min_ = −0.21 e Å^−^^3^

### Special details

**Table:**

Geometry. All e.s.d.'s (except the e.s.d. in the dihedral angle between two l.s. planes) are estimated using the full covariance matrix. The cell e.s.d.'s are taken into account individually in the estimation of e.s.d.'s in distances, angles and torsion angles; correlations between e.s.d.'s in cell parameters are only used when they are defined by crystal symmetry. An approximate (isotropic) treatment of cell e.s.d.'s is used for estimating e.s.d.'s involving l.s. planes.
Refinement. Refinement of *F*^2^ against ALL reflections. The weighted *R*-factor *wR* and goodness of fit *S* are based on *F*^2^, conventional *R*-factors *R* are based on *F*, with *F* set to zero for negative *F*^2^. The threshold expression of *F*^2^ > σ(*F*^2^) is used only for calculating *R*-factors(gt) *etc*. and is not relevant to the choice of reflections for refinement. *R*-factors based on *F*^2^ are statistically about twice as large as those based on *F*, and *R*- factors based on ALL data will be even larger.

### Fractional atomic coordinates and isotropic or equivalent isotropic displacement parameters (Å^2^)

**Table:**

	*x*	*y*	*z*	*U*_iso_*/*U*_eq_	
S1	0.25538 (5)	0.86234 (4)	0.23201 (4)	0.05546 (19)	
O1	0.36858 (17)	0.78724 (15)	0.26990 (10)	0.0777 (5)	
O2	0.29533 (18)	0.97863 (13)	0.21357 (12)	0.0803 (5)	
N1	0.10568 (17)	0.86333 (13)	0.29521 (10)	0.0483 (4)	
C1	0.1958 (2)	0.80108 (17)	0.13244 (12)	0.0480 (4)	
C2	0.1431 (3)	0.8704 (2)	0.06558 (16)	0.0702 (6)	
H2	0.1435	0.9495	0.0718	0.084*	
C3	0.0896 (3)	0.8205 (3)	−0.01092 (16)	0.0908 (9)	
H3	0.0530	0.8666	−0.0559	0.109*	
C4	0.0904 (3)	0.7056 (3)	−0.02042 (18)	0.0919 (9)	
H4	0.0546	0.6731	−0.0719	0.110*	
C5	0.1431 (3)	0.6373 (2)	0.04474 (18)	0.0800 (7)	
H5	0.1438	0.5584	0.0373	0.096*	
C6	0.1956 (2)	0.68386 (18)	0.12180 (14)	0.0579 (5)	
H6	0.2307	0.6365	0.1664	0.070*	
C7	−0.01977 (19)	0.94843 (16)	0.27965 (11)	0.0458 (4)	
H7	0.0252	1.0101	0.2449	0.055*	
C8	−0.0737 (2)	1.00139 (17)	0.36414 (12)	0.0535 (5)	
H8A	−0.1183	0.9425	0.4006	0.064*	
H8B	0.0154	1.0342	0.3947	0.064*	
C9	−0.1949 (3)	1.09394 (19)	0.34699 (16)	0.0680 (6)	
H9A	−0.2317	1.1236	0.4018	0.082*	
H9B	−0.1467	1.1565	0.3157	0.082*	
C10	−0.3316 (2)	1.0492 (2)	0.29526 (14)	0.0630 (6)	
H10A	−0.4030	1.1116	0.2825	0.076*	
H10B	−0.3871	0.9929	0.3293	0.076*	
C11	−0.2801 (3)	0.9953 (2)	0.21182 (14)	0.0703 (6)	
H11A	−0.2348	1.0533	0.1749	0.084*	
H11B	−0.3702	0.9632	0.1818	0.084*	
C12	−0.1603 (2)	0.9014 (2)	0.22887 (14)	0.0613 (6)	
H12A	−0.1255	0.8701	0.1742	0.074*	
H12B	−0.2084	0.8401	0.2615	0.074*	
C13	0.0697 (2)	0.75885 (17)	0.34458 (13)	0.0545 (5)	
H13A	0.1140	0.6935	0.3149	0.065*	
H13B	−0.0428	0.7487	0.3456	0.065*	
C14	0.1314 (3)	0.7611 (2)	0.43649 (16)	0.0804 (7)	
H14A	0.2445	0.7669	0.4357	0.097*	
H14B	0.0914	0.8284	0.4655	0.097*	
C15	0.0861 (4)	0.6566 (2)	0.48662 (18)	0.0980 (9)	
H15A	−0.0250	0.6564	0.4950	0.147*	
H15B	0.1385	0.6571	0.5419	0.147*	
H15C	0.1157	0.5894	0.4551	0.147*	

### Atomic displacement parameters (Å^2^)

**Table:**

	*U*^11^	*U*^22^	*U*^33^	*U*^12^	*U*^13^	*U*^23^
S1	0.0365 (3)	0.0626 (3)	0.0673 (4)	−0.0039 (2)	0.0035 (2)	−0.0127 (3)
O1	0.0456 (8)	0.1083 (13)	0.0789 (11)	0.0169 (8)	−0.0108 (7)	−0.0136 (9)
O2	0.0622 (9)	0.0621 (10)	0.1173 (13)	−0.0246 (7)	0.0279 (9)	−0.0219 (9)
N1	0.0423 (8)	0.0550 (9)	0.0477 (9)	0.0016 (7)	0.0022 (7)	−0.0050 (7)
C1	0.0392 (9)	0.0537 (11)	0.0514 (11)	0.0011 (8)	0.0112 (8)	0.0023 (9)
C2	0.0690 (15)	0.0709 (15)	0.0711 (16)	0.0122 (11)	0.0181 (12)	0.0127 (12)
C3	0.0806 (18)	0.140 (3)	0.0521 (15)	0.0295 (18)	0.0081 (12)	0.0178 (16)
C4	0.0700 (16)	0.142 (3)	0.0642 (17)	0.0134 (18)	0.0044 (13)	−0.0290 (18)
C5	0.0737 (17)	0.0819 (17)	0.0847 (19)	−0.0037 (13)	0.0127 (14)	−0.0285 (15)
C6	0.0567 (12)	0.0565 (12)	0.0607 (13)	0.0038 (9)	0.0086 (10)	−0.0045 (10)
C7	0.0402 (9)	0.0529 (11)	0.0444 (10)	−0.0004 (8)	0.0011 (7)	−0.0036 (8)
C8	0.0516 (11)	0.0602 (12)	0.0485 (11)	0.0030 (9)	−0.0064 (8)	−0.0130 (9)
C9	0.0713 (15)	0.0626 (13)	0.0702 (14)	0.0163 (11)	0.0034 (11)	−0.0114 (11)
C10	0.0527 (12)	0.0780 (14)	0.0584 (13)	0.0185 (10)	0.0022 (10)	0.0054 (11)
C11	0.0524 (12)	0.1059 (19)	0.0525 (12)	0.0112 (12)	−0.0068 (10)	0.0014 (12)
C12	0.0464 (11)	0.0857 (15)	0.0518 (12)	0.0056 (10)	−0.0062 (9)	−0.0209 (11)
C13	0.0507 (11)	0.0565 (12)	0.0564 (12)	0.0007 (9)	−0.0040 (9)	−0.0079 (9)
C14	0.0900 (18)	0.0855 (17)	0.0652 (15)	0.0007 (14)	−0.0233 (13)	0.0073 (13)
C15	0.119 (2)	0.095 (2)	0.0801 (18)	0.0125 (17)	0.0040 (17)	0.0243 (15)

### Geometric parameters (Å, °)

**Table:**

S1—O1	1.4274 (16)	C8—H8B	0.9700
S1—O2	1.4308 (16)	C9—C10	1.502 (3)
S1—N1	1.6187 (15)	C9—H9A	0.9700
S1—C1	1.7658 (19)	C9—H9B	0.9700
N1—C13	1.474 (2)	C10—C11	1.504 (3)
N1—C7	1.481 (2)	C10—H10A	0.9700
C1—C6	1.380 (3)	C10—H10B	0.9700
C1—C2	1.384 (3)	C11—C12	1.522 (3)
C2—C3	1.390 (4)	C11—H11A	0.9700
C2—H2	0.9300	C11—H11B	0.9700
C3—C4	1.351 (4)	C12—H12A	0.9700
C3—H3	0.9300	C12—H12B	0.9700
C4—C5	1.357 (4)	C13—C14	1.509 (3)
C4—H4	0.9300	C13—H13A	0.9700
C5—C6	1.379 (3)	C13—H13B	0.9700
C5—H5	0.9300	C14—C15	1.499 (3)
C6—H6	0.9300	C14—H14A	0.9700
C7—C8	1.520 (2)	C14—H14B	0.9700
C7—C12	1.530 (2)	C15—H15A	0.9600
C7—H7	0.9800	C15—H15B	0.9600
C8—C9	1.520 (3)	C15—H15C	0.9600
C8—H8A	0.9700		
			
O1—S1—O2	120.22 (11)	C8—C9—H9A	109.3
O1—S1—N1	107.15 (9)	C10—C9—H9B	109.3
O2—S1—N1	107.79 (9)	C8—C9—H9B	109.3
O1—S1—C1	107.16 (9)	H9A—C9—H9B	107.9
O2—S1—C1	106.21 (10)	C9—C10—C11	111.57 (18)
N1—S1—C1	107.79 (8)	C9—C10—H10A	109.3
C13—N1—C7	119.02 (15)	C11—C10—H10A	109.3
C13—N1—S1	118.51 (13)	C9—C10—H10B	109.3
C7—N1—S1	118.92 (12)	C11—C10—H10B	109.3
C6—C1—C2	119.5 (2)	H10A—C10—H10B	108.0
C6—C1—S1	120.41 (15)	C10—C11—C12	110.90 (17)
C2—C1—S1	120.04 (17)	C10—C11—H11A	109.5
C1—C2—C3	119.4 (2)	C12—C11—H11A	109.5
C1—C2—H2	120.3	C10—C11—H11B	109.5
C3—C2—H2	120.3	C12—C11—H11B	109.5
C4—C3—C2	120.5 (3)	H11A—C11—H11B	108.0
C4—C3—H3	119.8	C11—C12—C7	110.64 (17)
C2—C3—H3	119.8	C11—C12—H12A	109.5
C3—C4—C5	120.4 (3)	C7—C12—H12A	109.5
C3—C4—H4	119.8	C11—C12—H12B	109.5
C5—C4—H4	119.8	C7—C12—H12B	109.5
C4—C5—C6	120.7 (3)	H12A—C12—H12B	108.1
C4—C5—H5	119.7	N1—C13—C14	113.53 (17)
C6—C5—H5	119.7	N1—C13—H13A	108.9
C5—C6—C1	119.6 (2)	C14—C13—H13A	108.9
C5—C6—H6	120.2	N1—C13—H13B	108.9
C1—C6—H6	120.2	C14—C13—H13B	108.9
N1—C7—C8	111.13 (14)	H13A—C13—H13B	107.7
N1—C7—C12	113.91 (15)	C15—C14—C13	112.4 (2)
C8—C7—C12	110.00 (15)	C15—C14—H14A	109.1
N1—C7—H7	107.2	C13—C14—H14A	109.1
C8—C7—H7	107.2	C15—C14—H14B	109.1
C12—C7—H7	107.2	C13—C14—H14B	109.1
C9—C8—C7	110.68 (16)	H14A—C14—H14B	107.8
C9—C8—H8A	109.5	C14—C15—H15A	109.5
C7—C8—H8A	109.5	C14—C15—H15B	109.5
C9—C8—H8B	109.5	H15A—C15—H15B	109.5
C7—C8—H8B	109.5	C14—C15—H15C	109.5
H8A—C8—H8B	108.1	H15A—C15—H15C	109.5
C10—C9—C8	111.73 (18)	H15B—C15—H15C	109.5
C10—C9—H9A	109.3		
			
O1—S1—N1—C13	−31.31 (16)	C2—C1—C6—C5	0.1 (3)
O2—S1—N1—C13	−162.01 (14)	S1—C1—C6—C5	177.52 (16)
C1—S1—N1—C13	83.73 (14)	C13—N1—C7—C8	64.7 (2)
O1—S1—N1—C7	170.12 (13)	S1—N1—C7—C8	−136.84 (14)
O2—S1—N1—C7	39.42 (16)	C13—N1—C7—C12	−60.2 (2)
C1—S1—N1—C7	−74.84 (15)	S1—N1—C7—C12	98.24 (17)
O1—S1—C1—C6	30.33 (18)	N1—C7—C8—C9	176.37 (16)
O2—S1—C1—C6	160.00 (15)	C12—C7—C8—C9	−56.5 (2)
N1—S1—C1—C6	−84.70 (16)	C7—C8—C9—C10	55.9 (2)
O1—S1—C1—C2	−152.24 (16)	C8—C9—C10—C11	−55.4 (3)
O2—S1—C1—C2	−22.57 (18)	C9—C10—C11—C12	55.7 (3)
N1—S1—C1—C2	92.73 (17)	C10—C11—C12—C7	−56.7 (3)
C6—C1—C2—C3	0.5 (3)	N1—C7—C12—C11	−177.24 (17)
S1—C1—C2—C3	−176.93 (17)	C8—C7—C12—C11	57.2 (2)
C1—C2—C3—C4	−0.6 (4)	C7—N1—C13—C14	−103.9 (2)
C2—C3—C4—C5	0.1 (4)	S1—N1—C13—C14	97.55 (19)
C3—C4—C5—C6	0.5 (4)	N1—C13—C14—C15	177.0 (2)
C4—C5—C6—C1	−0.6 (3)		
